# Analyses of pathological cranial ultrasound findings in neonates that fall outside recent indication guidelines: results of a population-based birth cohort: survey of neonates in Pommerania (SNiP-study)

**DOI:** 10.1186/s12887-019-1843-6

**Published:** 2019-12-05

**Authors:** Judith Weise, Matthias Heckmann, Hagen Bahlmann, Till Ittermann, Heike Allenberg, Grzegorz Domanski, Anja Erika Lange

**Affiliations:** 1 Dept. of Neonatology & Paediatric Intensive Care Medicine, University Greifswald, F.-Sauerbruchstr, 17475 Greifswald, Germany; 2 Institute of Community Medicine, Div. of Health Care Epidemiology and Community Health, University Greifswald, Greifswald, Germany

## Abstract

**Background:**

Recent guidelines recommend a cranial ultrasound (CU) in neonates born at < 30 weeks gestation, admitted to the neonatal intensive care unit (NICU), or with a CU indication. Here, we addressed the need to extend these recommendations.

**Methods:**

We retrospectively reviewed 5107 CUs acquired in the population-based Survey of Neonates in Pomerania, conducted in 2002 to 2008. Neonates with conspicuous CUs that were ≥ 30 weeks gestation without recent indications for CU were identified and assigned to the following groups: with (I) or without (II) admission to neonatal care. We designated CU conspicuities as mild (MC) or significant (SC), and we investigated related neurodevelopment during follow-up.

**Results:**

Of 5107 neonates, 5064 were born at ≥30 weeks gestation and of those, 4306 received CUs without any indication for this examination. We found conspicuities in 7.7% (*n* = 47/610) of group I (*n* = 30 MC, *n* = 17 SC), and 3.2% (*n* = 117/3696) of group II (*n* = 100 MC, n = 17 SC). In group II, SC comprised, e.g., bilateral cysts, partial agenesis of the corpus callosum, and periventricular leukomalacia. Follow-up was available in 75% of infants in group II with MCs and SCs; of these, 12.8% had an abnormal neurological follow-up.

**Conclusions:**

We detected a high number of conspicuities in neonates without a CU indication. However, we could not demonstrate that ultrasound findings were associated with the neurological follow-up or any advantage to an earlier diagnosis. Our data did not support extending current guidelines or a general CU screening policy for all neonates.

## Background

The cranial ultrasound (CU) is a cost-effective, portable, non-invasive examination that does not require radiation. These features make it a valuable tool for assessing infantile brain structures [1]. The CU can detect cerebral pathologies, like hemorrhages or ventricle system disorders, without sedation [2]. Furthermore, early detection of these lesions ensures appropriate medical management and long-term assessments of neurodevelopmental disabilities [3].

Currently, the Quality Standards Subcommittee of the American Academy of Neurology and the Practice Committee of the Child Neurology Science recommend routine CU screening for all neonates < 30 weeks of gestational age (weeks GA) [3]. Beyond those recommendations, Leijser et al. claimed to include neonates that were admitted to the neonatal care unit (NCU) after birth [4]. The American Institute of Ultrasound in Medicine (AIUM) Practice Guidelines for the Performance of Neurosonography in Neonates and Infants 2014 also recommend CU for all infants with indications (Table 1) [5]. In Germany, neonatal screening for the auditory system, hip dysplasia, and metabolic diseases, but not CU, are included in regular check-ups (“U2/U3”) to ensure the early detection of treatable diseases with severe outcomes.

**Table 1:** Established AIUM indications for a neonatal CU and corresponding variables as predictors measured in the SNiP study [5]

AIUM Indications for CU in neonates	corresponding variables in the SNiP study
Hemorrhage or parenchymal abnormalities in preterm and term infants	birthweight < 1500 g and < 30 WG [3], vacuum or forceps delivery [8], systemic hypertension or hypotension, or perinatal asphyxia [9]
Hydrocephalus	signs (e.g., macrocephaly, curved fontanel, split cranial sutures) [10]
Vascular abnormalities	prenatal diagnosis (e.g., vascular malformations, aneurysms)
Possible or suspected hypoxic ischemic encephalopathy	symptoms (e.g., seizures, hypotonia, coma, respiratory distress), UA pH < 7.0, APGAR value of 0–3 at > 5 min [3]
Congenital malformations	prenatal diagnoses (e.g., cysts, cerebellar hypoplasia) [11], chromosomal anomalies or malformations, or metabolic diseases [12]
Congenital or acquired brain infections	mycoplasma, toxoplasmosis, cytomegalovirus, streptococcus
Signs and/or symptoms of central nervous system disorders	symptoms (e.g., facial malformations, macrocephaly, microcephaly, intrauterine growth restriction) [5]
Trauma	cephalo/subgaleal hematoma, subdural hematoma, subarachnoidal hemorrhage
Craniosynostosis	craniosynostosis
Previously documented abnormalities, including prenatal abnormalities	prenatal diagnoses (e.g., partial/complete agenesis of corpus callosum)
Patients treated with hypothermia, ECMO, or other support systems	hypothermia, ECMO etc.

AIUM: The American Institute of Ultrasound in Medicine; CU: cerebral ultrasound; SNiP: Survey of Neonates in Pomerania; WG: weeks of gestation; UA pH: umbilical arterial cord blood pH; APGAR score: assessment score for appearance, pulse, grimace, activity, and respiration of the neonate; ECMO: extra corporal membrane oxygenation

Additionally, assessments of fetal brain structures and possible deviations could be conducted with a prenatal ultrasound. Like CU, this prenatal examination is not generally performed in Germany, but recently, it has been offered regularly in the study region in western Pomerania.

The population-based birth cohort study, known as the Survey of Neonates in Pomerania (SNiP), included a CU for all participants, irrespective of indication, gestational age, or inpatient admission [6]. Here, we retrospectively reviewed those data. We aimed to determine the prevalence, severity, and outcome of cerebral conspicuities diagnosed in neonates, when there was no CU indication, according to conventional guidelines; i.e., we addressed the need for an extension of recent CU recommendations.

## Methods

### Study design

For the present analysis, we acquired data from the population-based prospective birth cohort study, SNiP, from 2002 to 2008 collecting data about morbidity and mortality of newborns and their mothers in a predefined region yielding to data about prevalence, risk factors and confounders for/of neonatal diseases. Based on this, cranial ultrasound was used to assess potential cerebral disorders. Physicians trained for this study collected data on newborn children and their mothers, regarding neonatal health, morbidity, and mortality. We calculated the prevalence rates for major neonatal diseases, risk factors, and confounding conditions, on a cross-sectional, prospective basis. All mothers with a complete data set were included, and they provided written informed consent to participate in the study.

The data collection was conducted as follows: (i) a standardized 5 to 10-min interview (84 variables), (ii) a self-administered questionnaire concerning socioeconomic background (40 variables), and (iii) a review of maternal medical records and the prenatal care booklet concerning the gestational period and any preventive examinations (149 variables). Details of the SNiP study have been reported previously by Ebner et al. [6] The collected data were anonymized and stored in an Access database. Approval for this study was obtained from the Ethics Committee of the Medical School of University. All analyses were based on complete data sets.

### Population

Current CU recommendations and the AIUM Practice Guidelines 2014 include indications: gestational age < 30 weeks GA, several diseases with correlation to neurodevelopmental disorders (see Table 1) [3, 5]. To evaluate the prevalence and severity of diagnoses in infants that fell outside the recent recommendations for CU, we identified all neonates with CU conspicuities that were born ≥30 weeks GA without indication, and grouped them as follows: (group I) neonates with admission and (group II) without admission to NCU after birth [5]. With respect to severity of illness, or degree of immaturity sick neonates were admitted to the neonatal care unit (special care baby unit or low dependency unit) to the intensive care unit (NICU). However, it was not possible to distinguish between these within the SNiP database. In general, all newborns of less than 34 weeks GA were admitted to special or intensive neonatal care based on severity of disease. Neonates ≥30 weeks GA with conspicuous lesions on the CU were additionally grouped by gestational maturity, as very/moderately preterm (30 to 34 weeks GA, *n* = 129) or late preterm/term (> 34 weeks GA, *n* = 4937).

### Examinations

The SNiP CU protocol was differentiated, with respect to weeks GA and admission: (i) CUs were conducted on days 1, 3, 7, and every 14 days for all neonates born < 30 weeks GA; (ii) CUs were conducted between the 30th and 36th weeks GA for neonates admitted to the NCU; and (iii) CUs were conducted between the 3rd and 10th day of life (check-up “E2”) for all other neonates. Consultants and registrars at the participating hospitals in Greifswald performed the CUs with a Sonolayer SSA-270A (TOSHIBA Medical Systems, Otawara, Japan), Acuson 128XP/10 Ultrasound System (Acuson, Mountain View, CA), the registrars at the participant hospital in Wolgast Picker CS 9100 EVB-405 (Bra Medik, HITACHI), and in Anklam Logia TM 200 (Pro Serius, Bangalore, India), and a 7.5 MHz sector array transducer; e.g., Wi Pro (GE Medical Systems, Bangalore, India). Afterwards, the findings were controlled by consultants or chief residents for Neonatology according to the hospital standards of University hospital, Greifswald. All CUs were performed in accordance with German performance guidelines [7].

An intrauterine ultrasound between the 19th to 22nd week of gestation was offered to all participants by German health insurance groups, as part of a prenatal prevention program, searching for malformations and especially brain abnormalities, which yielded to e.g. changes of the biparietal, frontooccipital diameter or head circumference. Next to this the prenatal prevention program contented ultrasounds between the 9th to 12th and 29th to 32nd week of gestation. Furthermore, all neonates were examined neurologically to evaluate tone, reflexes, and posture, during two regular check-ups (“U1/U2”) within the first 10 days of life. These examinations were not part of the SNiP protocol, but results were recorded in the SNiP database. When cerebral conspicuities were detected, the parents were given information about the necessity of a follow-up and the potential long-term outcome of the conspicuities found.

### Analyses

Associated risk factors, signs, and symptoms that were considered indications for CU, according to the AIUM Practice Guidelines, were related to variables in the SNiP database (Table 1). This procedure yielded the pre-analysis of neonates with indication for CU and classification into groups I and II.

For this study, all CU images with pathological findings were re-evaluated by one experienced neonatologist (A.E.L.). Pathological findings were classified as mild (MC) or significant (SC) conspicuities (Table 2). Furthermore, normal variants or undocumented cases in patient’s file or SNiP data base, which were marked as conspicuous in the primary database, could be excluded in this step to reduce the false positive rate of abnormal findings. We assessed long-term outcomes of neonates with MCs and SCs in the CUs. The University hospital, Greifswald is the only location in the study region with specialists for pediatric neurology and diagnostic and therapeutic interventions for neurodevelopmental disorders. When possible, we searched the medical records until recent days, with the following keywords: delay or disorder of motor and language development, learning disorders, hemiplegia, mental retardation, cerebral palsy, epilepsy, or diagnosis-related neonatal death. We analyzed records of neonates without a recent indication for CU or a NCU admission to investigate significant correlations with the following pre-, peri-, and postnatal predictors: infection with mycoplasma, streptococci, toxoplasmosis or cytomegalovirus, steroid administration in pregnancy for prevention of respiratory distress syndrome, chronic maternal condition, vacuum or forceps delivery, birth risks, APGAR score < 7 at 5 min (assessment score for appearance, pulse, grimace, activity, and respiration of the neonate), arterial umbilical cord blood pH < 7.0, <10th or > 90th percentile birth weight, micro- or macrocephalus, postnatal hypoglycemia, infection (e.g., *Escherichia. coli*), or respiratory distress.

**Table 2:** Classification of CU findings into significant or mild conspicuities, according to pathological neurodevelopmental sequelae

Cranial ultrasound	Neurodevelopmental outcome in the literature
Significant conspicuities	
Corpus callosum malformation	Mild behavioral problems to severe neurological disorders (e.g., autistic behavior), associated genetic syndromes, aneuploidies, malformations, inborn errors of metabolism [13–15]
Bilateral/multiple cyst (2–5 bilateral and/or unilateral cysts (max. 0.3–1.5 cm))	Congenital infection or genetic anomaly [16]
IVH, II-IV°	Epileptic disorders, perceptual difficulties, cognitive deficiencies, mental handicaps [10, 16, 17]
Hydrocephalus	Associated congenital brain anomalies, post-hemorrhagic, infection with neuromotor disorders, hearing loss, blindness, epilepsy [18, 19]
Periventricular leukomalacia (> 5 cysts > 0.3 cm, along the corpus callosum**)**	Global delay in myelination correlated with cerebral palsy and cognitive/behavioral abnormalities [10, 20]
Mild conspicuities	
Ventricular asymmetry/enlargement (> 0.5 cm difference of vertical distance between sulcus thalamicus and corpus callosum in two sagittal views at the level of the plexus choroideus in the lateral ventricles)	Normal variants or variants associated with autism, attention deficit hyperactivity disorder, learning disorders [22,23,21]
Unilateral/singular cyst	No screening necessary [16]
Increasing echogenicity	Physiologic immaturity of myelination of preterm infants or associated with hemorrhages, edemas etc. (follow-up necessary) [19]
IVH I°	No increase of conspicuous neurological impairment [18]

CU: cranial ultrasound, IVH: intraventricular hemorrhage

### Statistics

All analyses were performed with SPSS® version 22 for Windows 7/10® (Microsoft Corporation). We used the Fisher’s exact test for comparing 3 × 3-tables for statistical significance, which was set to a *p*-value < 0.05. Continuous data were expressed as the median to avoid excess influence from extreme values (< or > 95% of a normal curve). Categorical data were described with absolute numbers and percentages.

## Results

### Cranial ultrasound in the study population

**Fig. 1:**
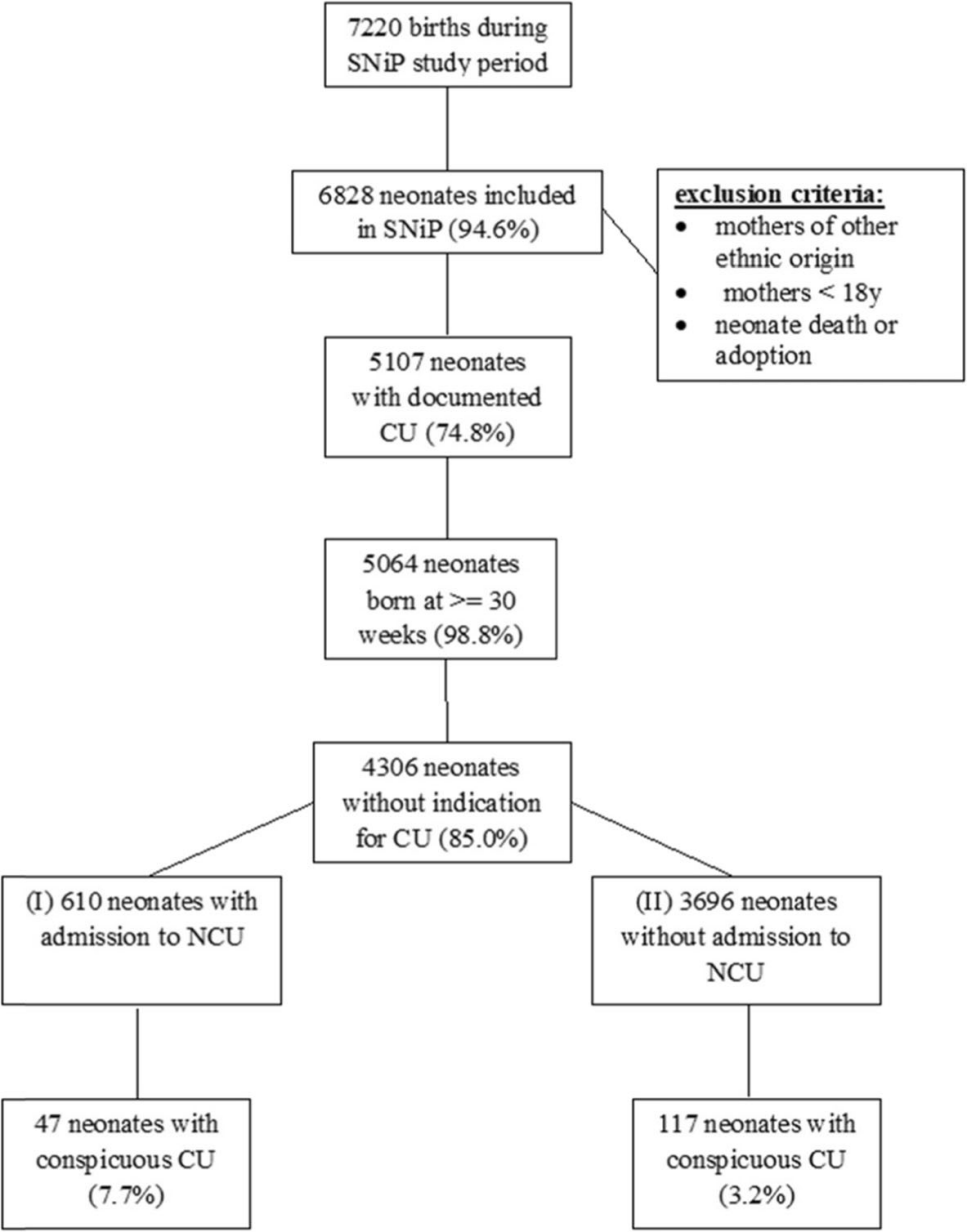
Flow chart of patient inclusion and grouping for analysis. SNiP: Survey of Neonates in Pomerania; CU: cerebral ultrasound; NCU: neonatal care unit; conspicuous CU: a potential lesion detected on the CU

In total, 7220 neonates were born in the study region during the evaluated period. Of these, 70.7% (*n* = 5107, median gestational age 39 weeks, median birth weight 3420 g) were included in the SNiP study (Fig. 1). Of these, 5107 infants received CUs, regardless of indication, gestational age, or admission. Error! Reference source not found. Shows the examined population according to predefined groups.

### Frequency of cranial ultrasound findings and classifications

In the primary database, 339/5107 (6.6%) ultrasound findings were classified as conspicuous. A re-evaluation of the stored ultrasound images was necessary to exclude normal variations, like Cavum septum pellucidum, to detect undocumented cases, and to assign conspicuities as either mild (MC) or significant (SC). This evaluation led to a reduction in the number of conspicuities (final *n* = 237; 4.6%) in the study population. To assess whether recent guidelines should be extended, we exclusively analyzed neonates born ≥30 weeks GA and without indication for CU (*n* = 4306).

758 neonates ≥30 weeks GA had indication for CU, based on AIUM Practice Guidelines. Of these, *n* = 58 (7.65%) had conspicuities in the CU.

4306 neonates had no indication for CU. Of these, 140 (3.3%) had a conspicuous CU (81.4% MC and 18.6% SC; Fig. 1, Table 3). Among 610 neonates without indication for CU but who were admitted to the NCU (group I), 7.7% (*n* = 47) had conspicuities in the CU. Among 3696 neonates with no indication who were not admitted to the NCU (group II), 3.2% (*n* = 117) had conspicuities in the CU. Details of MC are given in Table 4 and details of SC are given in Table 5 (Table 4, Table 5). All neonates with significant conspicuous CU had inconspicuous prenatal ultrasounds between the 19th and 22nd week. All neonates with significant conspicuous CU had controlled per MRI brain scan.

**Table 3:** Frequency of conspicuous cranial ultrasound findings in neonates > = 30 weeks of gestation without indication for cranial ultrasound

Cranial ultrasound groups	N of subgroup n/ N of group (%)
Conspicuities detected	140/3809 (3.7)
at ages 30–34 weeks gestation	4/12 (33.3)
at ages > 34 weeks gestation	136/3797 (3.6)
AND NCU admission (group I)	47/610 (7.7)
mild	30 (4.9)
significant	17 (2.8)
WITHOUT NCU admission (group II)	117/3696 (3.2)
mild	100 (2.7)
significant	17 (0.5)

Data are the number (%). NCU: neonatal care unit

**Table 4:** Frequency of mild conspicuities on cerebral ultrasound distributed among different gestational ages and groups

Gestational age group type of conspicuity	Group I	Group II
	Total cases	
30–34 weeks GA	**3**	**–**
unilateral singular cyst	2	
ventricular enlargement/asymmetry*	1	
> 34 weeks GA	**27**	**100**
ventricular enlargement/asymmetry*	12	55
unilateral singular parenchymal cyst	9	38
IVH I°	1	4
increased echogenicity	5	3
caudothalamic groove		1
thalamic		1
paraventricular	4	1
frontotemporal	1	

Data are the number of infants in each group. Weeks GA: weeks of gestational age; IVH: intraventricular hemorrhage, *measurement: > 0.5 cm vertical distance difference between the sulcus thalamicus and the corpus callosum in two sagittal views at the level of the plexus choroideus in the lateral ventricles

**Table 5:** Frequency of significant conspicuities on cerebral ultrasound distributed among different gestational age and groups

Gestational age group type of conspicuity	Group I	Group II
	Total cases	
30–34 weeks GA bilateral/multiple cyst	**1** 1	**–**
> 34 weeks GA	**16**	**17**
bilateral/multiple cyst	8	13
IVH II°	2	2
PVL	3	1
partial agenesis of corpus callosum	1	1
Arnold-Chiari malformation type I	1	
extracerebral space enlargement (> 1 cm) (cerebral atrophy)	1	

Data are the number of infants in each group. Weeks GA: weeks of gestational age; IVH: intraventricular hemorrhage; PVL: periventricular leukomalacia, with > 5 cysts > 0.3-cm in diameter, along the corpus callosum; bilateral/multiple cyst: 2–5 bilateral and/or unilateral cysts (max. 0.3–1.5 cm in diameter) [20].

### Risk factors for pathologic findings in CU

The analysis of pre-, peri- and post-natal risk factors for conspicuous CU in neonates without a NCU admission or an indication for CU (II) yielded no significant risk factors for this group. Documented cases of named predictors are shown in Table 6 (Table 6).

**Table 6:** Univariate Analysis of potential predictors of conspicuities detected in cranial ultrasounds performed in neonates without NCU admission or indication for CU (Group II)

Potential predictor	All cases (*n* = 3696)	Cases with mild conspicuities (n = 100)	Cases with significant conspicuities (n = 17)	*p* value*
APGAR < 7 at > 5 min	6 (0.2)	1 (1.0)	0	0.176
birth risks	1147 (31.0)	35 (35.0)	5 (29.4)	0.671
birth weight				0.163
< 10th percentile	111 (3.0)	1 (1.0)	0	
> 90th percentile	182 (4.9)	4 (4.0)	3 (17.6)	
delivery mode				
spontaneous	3297 (89.2)	88 (88.0)	14 (82.4)	
missed	399 (10.8)	12 (12.0)	3 (17.6)	
maternal chron. Disease	1154 (31.2)	38 (38.0)	5 (29.4)	0.133
RDS prophylaxis	109 (2.9)	6 (6.0)	0	0.200

Data are the number of individuals (%) in each group. *Mild or significant conspicuities were compared to inconspicuous cranial ultrasounds. NCU: neonatal care unit; CU: cranial ultrasound; APGAR score: assessment score for appearance, pulse, grimace, activity, and respiration of the neonate; RDS: respiratory distress syndrome. Birth risks (based on documentation in German maternal booklet for prenatal examinations): e.g., multiple births, vaginal infections, twin births etc. *P*-values were derived with Fisher’s Exact Test

### Long-term follow-up after conspicuous cerebral ultrasound

We investigated the documentation of neurodevelopmental follow-ups for all neonates with conspicuous CUs. These patients received at least a second CU scan to assess progression, stability, or regression of MCs and SCs. Of 140 neonates, 99 were further seen, examined or treated by the medical staff of the University Hospital, Greifswald (missing data group I: *n* = 12/47; group II: *n* = 29/117). Of the 99 patients monitored, 17 with MCs and 7 with SCs had the necessity of neurodevelopmental follow-ups (group I: *n* = 4 MCs, *n* = 5 SCs; group II: *n* = 13 MCs, n = 2 SCs). A significantly higher proportion of neurodevelopmental disorders was found among neonates with SCs (23.1%) compared to those with MCs (9.7%; *p* < 0.05 in group I and II). Of all neonates with admission to NCU (group I), 14.3% (*n* = 6/42) had a neurodevelopmental disorder. Neurodevelopmental disorders were found in 11.5% (*n* = 15/117) of patients in group II, and the cumulative incidence was 0.4% (Table 7). All of these neonates had negative microbiological blood tests for mycoplasma, cytomegalovirus, streptococcus, and toxoplasmosis. Neonates with partial agenesis of the corpus callosum (n = 1) and intraventricular hemorrhage II° (IVH; n = 2) were born with normal birth weights and APGAR scores. These three neonates showed no neurodevelopmental impairment related to a conspicuous CU during follow-up.

**Table 7:** Neurodevelopmental outcome in neonates with significant (n = 2) and mild (n = 13) conspicuities detected in the CU, despite no AIUM indication or NCU admission (group II)

Case	Gestational age (wks)	Birth weight (g)	APGAR scores^*^	CU findings	Neurodevelopment
101	41	3510	9/10/10	bilateral multiple cyst	speaking/language disorder
3672	39	3600	9/10/10	bilateral multiple cyst	delayed speaking development
2735	40	3680	9/9/9	ventricular enlargement	speaking/language disorder
1614	40	4270	9/10/10	ventricular asymmetry	speaking/language disorder
3048	41	3200	9/10/10	ventricular asymmetry	AD(H)D
744	40	4230	8/10/10	unilateral cyst in caudothalamic groove	auditory perception disorder
4708	39	2890	8/9/9	ventricular enlargement	tic disorder
2144	41	3900	7/9/10	ventricular asymmetry	epilepsy
3845	40	4220	9/10/10	ventricular enlargement	Rolando epilepsy, AD(H)D
2546	40	3940	9/9/10	ventricular asymmetry	AD(H)D
3466	40	3360	9/10/10	unilateral cyst in plexus	speaking disorder
3935	36	2960	9/10/10	unilateral cyst in plexus	delayed motor and speaking development
4899	39	4060	9/10/10	IVH I°	auditory perception disorder
5373	41	3810	8/9/10	unilateral cyst in septum pellucidum	adaptive functioning emotional disorder/tic disorder
3897	39	2825	8/9/10	unilateral cyst in plexus	delayed motor development

*APGAR scores were measured at 1 min/5 min/ > 5 min after birth; CU: cerebral ultrasound; AIUM: American Institute of Ultrasound in Medicine; NCU: neonatal care unit; APGAR score: assessment score for appearance, pulse, grimace, activity, and respiration of the neonate; AD(H)D: attention deficit (and hyperactivity) disorder; IVH: intraventricular hemorrhage

## Discussion

Based on recent international guidelines, a neonatal CU is recommended for the following defined risk neonatal groups: aged < 30 weeks GA or an indication for CU [3, 5]. Additionally, a proposal was made for neonates with admission to NCU [4]. In this study, we re-evaluated these recommendations to determine whether an extension was warranted. Specifically, we addressed whether performing a CU was justified for all infants, irrespective of indications, based on the prevalence and severity of CU conspicuities and their neurodevelopmental outcomes. Our population comprised 3696 neonates that had no indication for CU and were not admitted to the NCU. We found that the cumulative incidence of a conspicuous CU finding in neonates without an indication or NCU admission was 3.2%, which was, as expected, lower than the incidences among neonates with indications (7.65%) and neonates with NCU admissions (7.7%). Significant anomalies, like corpus callosum hypoplasia, periventricular leukomalacia (PVL), and IVH II°, were not associated with a conspicuous neurological follow-up; thus, we lacked a correlation between conspicuous CU findings and neurological impairments [8, 9]. Among the 117 neonates without an indication for CU, but with CU conspicuities, 85.5% had MCs and 14.5% had SCs. Ballardini et al. postulated a prevalence of 1.7% for conspicuous CUs in asymptomatic neonates aged > 37 weeks GA, and the distribution of MCs and SCs was similar to that in the present study [10]. The prevalence of SCs was 0.33% in group II, which was higher than the prevalence of significant anomalies found by Ballardini et al. (0.19%) or that found by Wang et al. (0.25%) [10, 11]. The inclusion of bilateral cysts as an SC yielded a higher prevalence in our study. Even higher prevalences were reported by Heibel et al. (9%) and Gover et al. (11.2%). Those values might be explained by differences in operators, populations, and/or techniques [8, 12]. None of the neonates with bilateral cysts had positive microbiological blood tests. Therefore, this study could not demonstrate that the probability of congenital infections increased with the presence of bilateral parenchymal cysts [8].

Most MCs (93%) in group II comprised ventricular asymmetry, mild ventricular enlargement, or unilateral singular cysts. Of these MCs, 13% (*n* = 13/100) were associated with later neurological impairments, like attention deficit (and hyperactivity) disorder, speaking/language disorders, or epilepsy. This finding did not suggest that the guidelines should be extended for these diagnoses, because, for example, single cysts had a negligible likelihood of leading to neurodevelopmental impairment, and thus, they did not require follow-up [8].A large proportion of SCs (76%) in group II comprised bilateral or multiple cysts. These cysts can be diagnosed easily with CU. One in 4–5 neonates with bilateral, multiple choroid plexus cysts or subependymal pseudocysts harbor a chromosomal anomaly, like trisomy 18, trisomy 21, Cri du chat syndrome, or Zellweger syndrome, or a congenital infection (cytomegalovirus or rubella virus) with the highest positive likelihood ratio [8]. Multiple cysts, irrespective of location, can be associated with, for example, IVH and PVL. In addition, multiple cysts can lead to further malformations that have an impact on neurodevelopment [13]. In this study, among patients in group II with SCs that represented bilateral cysts, 11.8% (*n* = 2/17) showed neurodevelopmental delay or speaking disorders; the cumulative incidence was 0.05% (n = 2/3696) in all neonates without a CU indication or NCU admission. None of the named associated diseases or malformations were found.

PVL typically appears in very premature infants, but it is also detected on a CU or magnetic resonance imaging (MRI) at an unknown incidence in late preterm and term infants [13]. PVL grades 1 to 3 exhibit (1) an abnormally high signal intensity, (2) loss of periventricular white matter, or (3) a necrotic, diffuse component, which can lead to cysts, focal scars, and global delay in myelination. These features were correlated with cerebral palsy and cognitive or behavioral abnormalities [13, 14]. Among children with PVL grade 1, 56% might also have minor motor problems. An association with West syndrome, which presents with relapsing seizures, increases the probability of disorders in brain development and psychomotor functioning [15]. In this study, PVL was not associated neurological impairment, and the cumulative incidence of PVL in group II was low (0.03%).

In the general population, the incidence of partial or complete agenesis of the corpus callosum is 0.3 to 0.7%, and it can be detected with CU [16]. The outcome of partial agenesis of the corpus callosum spans a large range, from mild behavioral problems to severe neurological disorders. Compared to complete agenesis, a partial agenesis is associated with a higher probability of combined genetic syndromes, aneuploidies, further cerebral malformations, inborn errors of metabolism, and extracerebral malformations [17–19]. .Compared to the intelligence of the general population, children with isolated corpus callosum agenesis have a higher probability of borderline intelligence and a lower probability of a Full Scale Intelligence Quotient ≥100 [20]. In this study, we found a cumulative incidence with isolated corpus callosum agenesis of 0.03% in group II, similar to that reported by Wang et al. (0.08%), but we found no correlation with neurological impairment [11].

IVH ≥ II° can induce epileptic disorders, perceptual difficulties, cognitive deficiencies, and mental handicaps [8, 14, 21]. In particular, IVH can induce a worse outcome in full-term neonates than in preterm neonates [14]. Even a mild IVH alone can independently have an impact on neurological outcome, compared to no IVH [22]. .We found a 0.05% cumulative incidence of IVH in group II, which was lower than the incidences found in studies by Gover (0.2%) and Wang et al. (0.1%), and we found no association with neurological impairment [11, 12].

Our results suggested that it is not necessary to extend the recent CU guidelines for neonates without a CU indication. We found an existing, but low cumulative incidence of mild and significant anomalies; thus, we could not demonstrate a correlation between ultrasound findings and neurological follow-up. Neurological development disorders can be detected during the regular, continual pediatric check-ups in Germany. Nevertheless, neonatologists, follow-up-clinicians, parents, and teachers must be cautious in assessing infants with the named pathologies, because additional resources and sustained rehabilitation might be necessary to maximize the child’s potential [20, 22].

Our study findings emphasized the necessity of conducting CUs in cases specified in the recommended guidelines. Indeed, we found that all neonates < 30 weeks GA had an indication for CU; the cumulative incidence was 34.9% for a CU finding, and the probability was 20.9% for neurodevelopmental disorders. Among neonates with a NCU admission (7.7%) the highest cumulative incidence for SCs was observed (2.8% SCs), and of these, 16.4% displayed neurodevelopmental disorders. Almost every 5th neonate born at 30–34 weeks GA had a CU conspicuity. This finding was consistent with findings by Ballardini et al., who suggested that CU should be performed in all neonates born at ≤34 weeks GA [9].

The major strength of this study was the large number of neonates (*n* = 5109) with data available from the population-based prospective cohort study, SNiP, including 3696 neonates without a CU indication The analysis included neonates at ages that ranged between 22 and 43 weeks GA, which exceeded the age ranges studied in previous investigations.

Although the SNiP database comprised 250 variables, data on follow-up were not included; consequently, those data were collected retrospectively. The major limitation of this study was the 25% loss to follow-up in the most interesting group of neonates, which had no indication for CU. The nature of the retrospective study design for the follow up and reevaluation of sonographies impeded a detailed assessment of developmental disorders; therefore, we might have missed some neonates with conspicuities in the CU that later displayed developmental disorders, which is a limitation. The increased diagnostic accuracy of prenatal ultrasound in the last decade can also increase the amount of prenatal diagnosed abnormal cerebral findings in future. Furthermore, any unusual CU findings, including normal variations, were documented as conspicuities in the SNiP database. To eliminate normal variations and detect missed conspicuities, we performed a retrospective re-evaluation of the scanned CU images; this re-evaluation might have resulted in some unrecognized pathologies.

## Conclusion

This study could not demonstrate either a probable association between ultrasound findings and neurological follow-ups or any advantage to performing an earlier diagnosis. Therefore, our data did not support an extension of current guidelines or the need for a general CU screening policy for all neonates. Nevertheless, the CU is helpful to detect brain abnormalities earlier. We recommend that CU should be performed in all neonates that conform to the recent recommendations of the AIUM, Leijser et al., Ment et al., and Ballardini et al. [3–5, 9] Further investigations of outcome are needed.

## Data Availability

The data of the SNIP-study is publicly available. This is a data repository where any researcher can register and find data dictionary as well as an online application tool for getting access to data. Upon an application by registered users, the Research Cooperation Community Medicine (RCC) of the University of Greifswald, Germany, which is funded by the Federal Ministry of Education and Research (grant no. ZZ 96030) decides on granting access to the data based on scientific guidelines.

## Abbreviations

AIUM: American Institute of Ultrasound in Medicine
APGAR score: Assessment score for appearance, pulse, grimace, activity, and respiration of the neonate
CU: Cranial ultrasound
IVH: Intraventricular hemorrhage
MC: Mild conspicuity
NCU: Neonatal care unit
PVL: Periventricular leukomalacia
SC: Significant conspicuity
SNiP: Survey of Neonates in Pomerania
weeks GA: Weeks of gestational age
